# A cognitive profile of multi-sensory imagery, memory and dreaming in aphantasia

**DOI:** 10.1038/s41598-020-65705-7

**Published:** 2020-06-22

**Authors:** Alexei J. Dawes, Rebecca Keogh, Thomas Andrillon, Joel Pearson

**Affiliations:** 1 School of Psychology, The University of New South Wales, Sydney, New South Wales, Australia; 2 School of Psychological Sciences and Turner Institute for Brain and Mental Health, Monash University, Melbourne, Victoria Australia

**Keywords:** Neuroscience, Psychology

## Abstract

For most people, visual imagery is an innate feature of many of our internal experiences, and appears to play a critical role in supporting core cognitive processes. Some individuals, however, lack the ability to voluntarily generate visual imagery altogether – a condition termed “aphantasia”. Recent research suggests that aphantasia is a condition defined by the absence of visual imagery, rather than a lack of metacognitive awareness of internal visual imagery. Here we further illustrate a cognitive “fingerprint” of aphantasia, demonstrating that compared to control participants with imagery ability, aphantasic individuals report decreased imagery in other sensory domains, although not all report a complete lack of multi-sensory imagery. They also report less vivid and phenomenologically rich autobiographical memories and imagined future scenarios, suggesting a constructive role for visual imagery in representing episodic events. Interestingly, aphantasic individuals report fewer and qualitatively impoverished dreams compared to controls. However, spatial abilities appear unaffected, and aphantasic individuals do not appear to be considerably protected against all forms of trauma symptomatology in response to stressful life events. Collectively, these data suggest that imagery may be a normative representational tool for wider cognitive processes, highlighting the large inter-individual variability that characterises our internal mental representations.

## Introduction

Visual imagery, or seeing with the mind’s eye, contributes to essential cognitive processes such as episodic memory^1^, future event prospection^2^, visual working memory^3^, and dreaming^4^. By allowing us to re-live the past and simulate hypothetical futures, visual imagery enables us to flexibly and adaptively interpret the events we experience in the world^5^, and by extension appears to be an important precursor to our ability to plan effectively and engage in guided decision-making. Consequently, the frequency and content of maladaptive visual imagery are often defining features of mental illness^6^ and mental imagery is often elevated in disorders characterised by hallucinations^7,8^.

One of the most significant findings to date is that despite the prevalence of visual imagery use in the wider population, and despite its functional utility in cognition, certain individuals lack the ability to visualise altogether – a condition recently termed “aphantasia”^9^. Beyond self-report measures, this condition is characterised by stark differences between individuals who can and cannot visualise on an objective measure of imagery’s sensory strength^10^. This suggests that rather than reflecting inaccurate phenomenological reports or poor population-specific metacognition, aphantasia appears to represent a veridical absence of voluntarily generated internal visual representations.

The potential impact of visual imagery absence on wider cognition remains unknown. No research to date has empirically verified whether this phenomenology extends to other internal experiences and mental processes. This presents us with a rare opportunity to extend a cognitive fingerprint of aphantasia, in order to better clarify the role of visual imagery in wider psychological functioning and explore the impact of its absence on the subjective lives of individuals with a “blind mind”. Here we investigated whether individuals with aphantasia report reduced imagery in other multi-sensory domains, and assessed self-reports of episodic memory ability and trauma symptomatology in response to stressful life events, in addition to reported mind-wandering frequency and dreaming phenomenology.

## Method

### Participants

We compared a group of self-identified aphantasic individuals with two independent control groups of individuals with self-reported intact visual imagery on a range of questionnaires. The current study was approved by the UNSW Human Research Ethics Advisory Panel (HREAP-C) in line with National Health and Medical Research Council (NHRMC) guidelines on ethical human research. All participants gave informed consent before completing the study.

Given the need for more research in this area, we sought to collect data on as many aphantasic participants as possible. With the limited number of previous studies on aphantasia using small sample sizes of *N* = 10–20^9,10^, it was difficult to estimate required sample sizes for our study based on these results alone. We nevertheless used the limited data available to derive approximate effect sizes for group differences in these studies in the range of *d* = 1.0–3.0. Effect sizes in small sample studies are often inflated, however, and we expected weaker effects across multiple comparisons in our study, especially in non-imagery domain comparisons. Establishing a comparatively moderate expected effect size of *d* = 0.5, with 80% power and a highly conservative alpha of 0.0002 (see Statistical Analyses in Methods), we estimated that at least 170 participants would be required in each comparison group. Because our study was easily accessible online and received more participant responses than anticipated within our data collection window, we exceeded our sample size aim (*N* = 170) and ceased data collection for our aphantasic participant group at the sample size reported below. We then collected an equivalent number of participants for our independent control groups. Sample sizes for the aphantasia group, control group 1 and control group 2 were approximately equal after data cleaning and exclusions (*n* = 267, *n* = 203 and *n* = 197, respectively).

#### Aphantasia group

Aphantasic individuals in our study were recruited from online community research platforms (https://www.facebook.com/sydneyaphantasiaresearch/) and participated in exchange for entry into a gift card prize draw. 317 aphantasic participants in total completed our study, of whom 33 participants were excluded from analysis due to missing data (not completing all questionnaires). An additional 17 participants were excluded from our aphantasic sample due to unclear reporting (e.g. scoring at ceiling on the Vividness of Visual Imagery Questionnaire (VVIQ; see Methods) in line with older versions of the scale that used reversed scoring compared to the current version of the scale). Our final sample of aphantasic individuals included for analysis contained 267 participants (48% females; mean age = 33.97 years, *SD* = 12.44, range = 17–75 years).

#### Control group 1 (MTurk)

Participants in our main control group were recruited using Amazon Mechanical Turk (MTurk) and were remunerated to complete the study. This main control group sample comprised of 205 participants, two of whom were excluded from final analysis due to study incompletion. Our final sample for our main control group thus consisted of 203 participants (35% females; mean age = 33.82 years, *SD* = 9.33, range = 20–70 years) who were matched on mean age with our aphantasic sample (mean age difference = 0.15 years, *p* = 0.89, BF_10_ = 0.107).

#### Control group 2 (Undergraduates)

A second control group of 193 first-year undergraduate psychology students were tested using the same experimental design. Participants in our second control group (73% females; mean age = 19.33 years, *SD* = 3.69, range = 17–55 years) completed the study in exchange for course credit. All participants were included in final analysis (see section titled Control Group 2: Replication Analysis, in Results).

### Aphantasia sample characteristics

#### Demographics

A table of sample demographics for all groups can be found in the Supplementary Information (see Table S1). Our sample population of aphantasic participants were recruited from online community research platforms dedicated to the topic of visual imagery ability and aphantasia. Both participants who did and didn’t identify with a history of visual imagery absence were invited to participate in the study. Of the 267 participants in our sample who reported aphantasia, a majority reported English as their first language (83%, *n* = 220) and identified as White/Caucasian (88%, *n* = 235). 31 countries of residence were listed, with a majority of participants originating from the United States of America.

#### Clinical history

Of the aphantasic sample, 24% of participants reported a history of mental illness (compared to 18% in control group 1; χ^2^_1,470_ = 3.644, *p* = 0.06), 1% reported a history of epilepsy or seizures (compared to 8% in control group 1; χ^2^_1,470_ = 14.881, *p* < 0.001), 4% reported a neurological condition (compared to 7% in control group 1; χ^2^_1,470_ = 1.765, *p* = 0.184), 9% reported having suffered head injury or trauma at least once (compared to 9% in control group 1; χ^2^_1,470_ = 0.019, *p* = 0.890), and 0.7% reported having once suffered a stroke (compared to 6% in control group 1; χ^2^_1,470_ = 10.634, *p* < 0.01).

#### Imagery scores

Weak visual imagery ability is typically defined by a total score of 32 or less on the Vividness of Visual Imagery Questionnaire (VVIQ: see Imagery Questionnaires in Materials), a five-point Likert self-report scale which ranges from 16–80^9,11^. A total score of 32 is equivalent to rating one’s agreement on every questionnaire item at 2 (“Vague and dim”). On average, aphantasic participants in our sample scored 17.94 on the VVIQ (including 70% with total floor scores of 16), compared to 58.12 in control group 1 (see Imagery Results section) and 58.79 in control group 2 (see Table S2 in Supplementary Information).

### Experimental procedure

Questionnaires were administered online using the Qualtrics research platform, and presented to each participant in random order. All participants completed a total of 206 questions in eight questionnaires. These questionnaires assessed self-reported multi-sensory imagery, episodic memory and future prospection, spatial abilities, mind-wandering and dreaming propensity, and response to stressful life events, as detailed below.

### Materials

#### Imagery questionnaires

The Vividness of Visual Imagery Questionnaire (VVIQ^11^; Marks, 1973) is a 16-item scale which asks participants to imagine a person as well as several scenes and rate the vividness of these mental images using a 5-point scale ranging from 1 (“No image at all, you only ‘know’ that you are thinking of the object”) to 5 (“Perfectly clear and <as> vivid as normal vision”). A single mean score on the VVIQ was computed for each participant. The Questionnaire upon Mental Imagery (QMI^12^; Sheehan, 1967) asks participants to rate the clarity and vividness of a range of imagined stimuli in seven sensory domains (visual, auditory, tactile, kinesthetic, taste, olfactory, emotion) on a 7-point scale ranging from 1 (“I think of it, but do not have an image before me”) to 7 (“Very vivid and as clear as reality”). There are 35 items on the QMI in total, with five items corresponding to each of the seven sensory domains. The Object and Spatial Imagery Questionnaire (OSIQ^13^; Blajenkova, Kozhevnikov, & Motes, 2006) is a 50-item scale which requires participants to indicate how well each of several statements on object imagery ability (e.g. “When I imagine the face of a friend, I have a perfectly clear and bright image”) and spatial imagery ability (e.g. “I am a good Tetris player”) applies to them on a 5-point scale ranging from 1 (“Totally disagree”) to 5 (“Totally agree”). There are 25 items each comprising the Object and Spatial imagery domains of the OSIQ, averaged to form a mean score on each domain.

#### Memory questionnaires

The Episodic Memory Imagery Questionnaire (EMIQ; on request) is a custom designed, 16-item self-report questionnaire which aims to assess the subjective vividness of episodic memory. Items on the EMIQ were partially derived from the VVIQ^11^ scale (Marks, 1973) and modified for context. The EMIQ asks participants to remember several events or scenes from their life and rate the vividness of these scenes using a 5-point scale ranging from 1 (“No image at all, I only ‘know’ that I am recalling the memory”) to 5 (“Perfectly clear and as vivid as normal vision”). A single mean score on the EMIQ was computed for each participant. The Survey of Autobiographical Memory (SAM^14^; Palombo, Williams, Abdi, & Levine, 2013) is a 26-item scale which measures participant agreement with a number of statements related to general episodic memory ability on a 5-point scale ranging from 1 (“Strongly disagree”) to 5 (“Strongly agree”). The scale is divided into 4 components: Event Memory (averaged across eight items, e.g. “When I remember events, in general I can recall people, what they looked like, or what they were wearing”), Future Events (averaged across six items; e.g. “When I imagine an event in the future, the event generates vivid mental images that are specific in time and place”), Factual Memory (averaged across six items; e.g. “I can learn and repeat facts easily, even if I don’t remember where I learned them”) and Spatial Memory (averaged across six items; e.g. “In general, my ability to navigate is better than most of my family/friends”).

#### Dreaming and daydreaming questionnaires

Part 1 of the Imaginal Process Inventory (IPI;^15,16^ Giambra, 1980; Singer & Antrobus, 1963) consists of 24 items which assess the self-reported frequency of day dreams (or mind-wandering episodes) and night dreams on a 5-point agreement scale which differs on each question (e.g. “I recall my night dreams vividly”, ranging from a) “Rarely or never” through to e) “Once a night”). The Subjective Experiences Rating Scale (SERS^17^; Kahan & Claudatos, 2016) comprises 39 questions which assess the qualitative content and subjective experience of participants’ night dreams generally (e.g. “During your dreams whilst asleep, <to what extent> do you experience colors”) on a 5-point rating scale ranging from 0 (“None”) to 4 (“A lot”). There are several sub-components of the scale which measure reported structural features of participants’ dreams (e.g. how bizarre one’s actions were, or how much perceived control participants experienced, during their dreams). The SERS is divided in our study into six dream components: Sensory, Affective, Cognitive, Spatial Complexity, Perspective and Lucidity. These components reflect typical SERS scale divisions, with the exception of Lucidity (in which we merge two existing components (Awareness and Control) of the previously published SERS scale^17^ in order to improve the readability of Fig. 2).

#### Trauma response questionnaire

The Post-Traumatic Stress Disorder (PTSD) Checklist for DSM-5 (PCL-5^18^; Weathers *et al*., 2013) measures self-reported responses to stressful life events. It asks participants to indicate how much they have been bothered by a problem related to a stressful life event on a 5-point scale ranging from 1 (“Not at all”) to 5 (“Extremely”). The PCL-5 contains 20 questions which are broken into four clinically relevant symptom categories: Intrusions (e.g. “Repeated, disturbing, and unwanted memories of the stressful experience”), Avoidance (e.g. “Avoiding memories, thoughts, or feelings related to the stressful experience”), Negative Alterations in Cognitions and Mood (e.g. “Blaming yourself or someone else for the stressful experience and what happened after it”), and Arousal and Reactivity (e.g. “Feeling jumpy or easily startled”). PTSD diagnosis can only be established by a professional practitioner in a structured clinical interview, and although cut-off scores on the PCL-5 are often used as an adjunct screening tool, the scale is not used for diagnostic purposes here.

### Statistical analyses

Non-parametric Mann-Whitney U hypothesis tests were conducted in SPSS 25.0 for Mac OS using Bonferroni adjusted alpha levels of *α* = 0.0002 (0.05/206 where 206 is the total number of question items across all questionnaires) to correct for multiple comparisons. Estimates of effect sizes *r* were computed using the following formula:

$$r=\frac{Z}{\sqrt{N}}$$

where *Z* is the Mann-Whitney standardized test statistic, *N* the total sample size of the combined groups, and *r* the output effect size estimate (comparable with Cohen’s *d* effect size interpretations^19^). Because we adopted a highly conservative adjusted alpha, Mann-Whitney tests were supplemented by Bayesian analyses conducted in JASP. For all Bayesian analyses, a Cauchy prior of 0.707 was used. Bayes factors were used to help compare the weight of evidence for between-group differences across test comparisons, whilst Mann-Whitney tests were used to make overall inferences about test direction and significance. Bayes factors were interpreted according to common threshold guidelines^20^, where 1 = “No evidence”, 1–3 = “Anecdotal evidence”, 3–10 = “Moderate evidence”, 10–30 = “Strong evidence”, 30–100 = “Very strong evidence”, and >100 = “Extreme evidence”.

#### Data transformation

**Figure 1:**
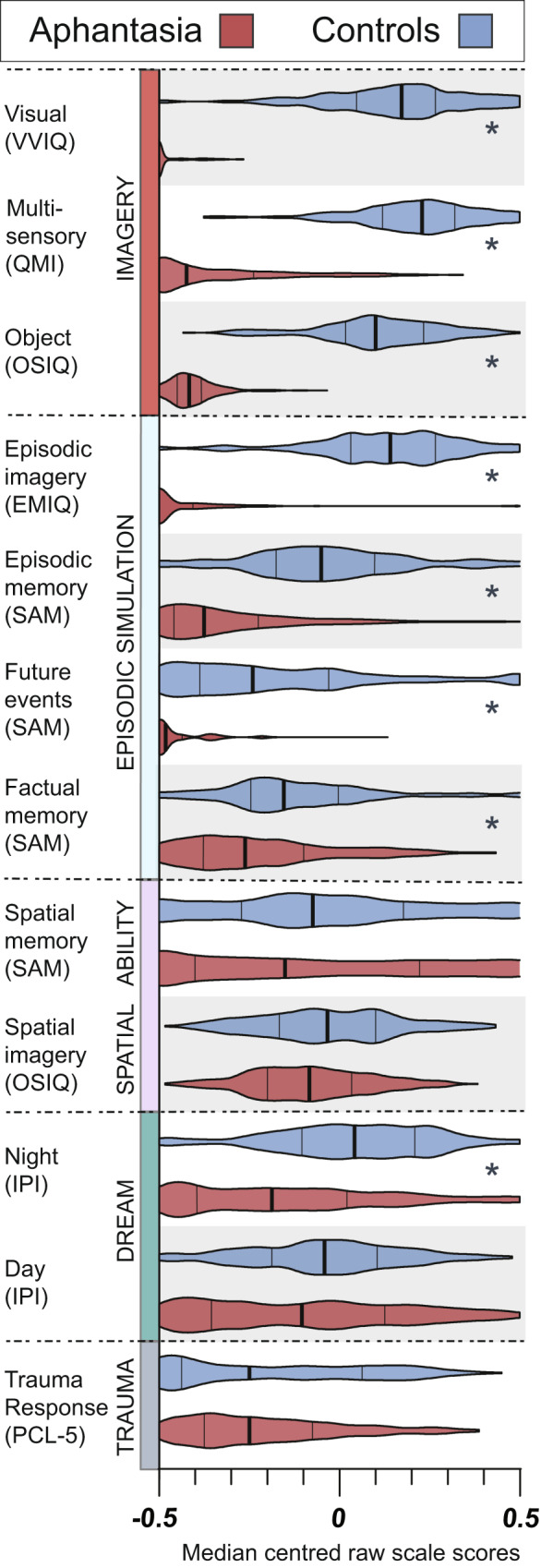
Summary of self-reported cognition questionnaires for individuals with aphantasia (red, *n* = 267) and control group 1 participants with visual imagery (blue, *n* = 203). Violin plots of median-centred scale scores with median (bold line), lower and upper quartiles (thin lines) and kernel density-smoothed frequency distribution (shaded area) coloured by group. Each pair of violin plots represents transformed raw data (see Data Transformation, Method). Stars to the right of group plot segments indicate Mann-Whitney test significance at threshold *p* < 0.0002.

All analyses were conducted on raw data. Data visualisation for Fig. 1 only, however, was carried out on median-centered raw questionnaire data using the following transformation:

$$y=\frac{x-\left(S.min+\frac{S.max-S.min}{2}\right)}{S.max-S.min}$$

where *y* is the transformed score; *x* the raw individual item score for scale *S*, and *S.min* and *S.max* the lowest and highest possible scores on that scale, respectively. This transformation allows us to graphically compare results across scales, with a value of −0*.5* representing the lowest possible score, 0 the median score, and *0.5* the maximum possible score on each scale.

## Hypotheses

We expected aphantasic individuals to report reduced visual imagery ability compared to controls, in line with previous findings^9,10^. There is some suggestion that auditory imagery may also be reduced in individuals who report visual imagery absence, however this evidence comes from case studies with limited sample sizes^1^. We therefore had no strong hypotheses regarding group differences in other multi-sensory imagery domains.

Given the proposed importance of mental imagery for the reliving of past life events^21^, we predicted that aphantasic individuals would report general alterations to episodic memory and future prospection processes, as well as reductions in episodic memory vividness.

Clinical research has traditionally placed heavy emphasis on the symptomatic role of visual imagery in mental health disorders including depression, social phobia, schizophrenia and post-traumatic stress disorder (PTSD), amongst others^6^. We therefore hypothesised that visual imagery absence might partially protect aphantasic individuals from experiencing some trauma symptomatology (such as vivid memory intrusions) in response to stressful past events.

Although neural measures suggest that dreaming is often characterised by vivid and objectively measurable internal visual experiences^4^, previous evidence on dreaming in aphantasia is somewhat inconclusive^22^. The overall impact of visual imagery absence on involuntary imagery processes (such as mind-wandering and dreaming whilst asleep) is therefore largely unclear, and we had no strong predictions regarding group differences in mind-wandering frequency, dream frequency, or dream phenomenology and content.

Lastly, we expected aphantasic self-reports of spatial imagery and spatial navigation abilities to align with data from previous studies suggesting that despite visual imagery absence, spatial abilities (as measured by questionnaires and performance on mental rotation and visuo-spatial tasks) appear to be largely preserved in aphantasia^10,22^.

## Results

The aim of the present study was to investigate the subjective impact of visual imagery absence on cognition. To achieve this, we compared self-reports of aphantasic individuals with those of general population individuals (with self-reported intact visual imagery) on several cognitive domains including multi-sensory imagery, episodic memory, trauma response, dreaming and daydreaming, and spatial abilities. The main results sections presented here all describe between-group tests comparing our aphantasic sample with our first control group of age-matched participants recruited from MTurk (see Tables S2–6 in Supplementary Information). For replication comparisons with our second control group sample of undergraduates, see section at end of Results titled “Control Group 2: Replication Analysis”.

### Control Group 1: Main Comparisons

#### Imagery results

**Figure 2:**
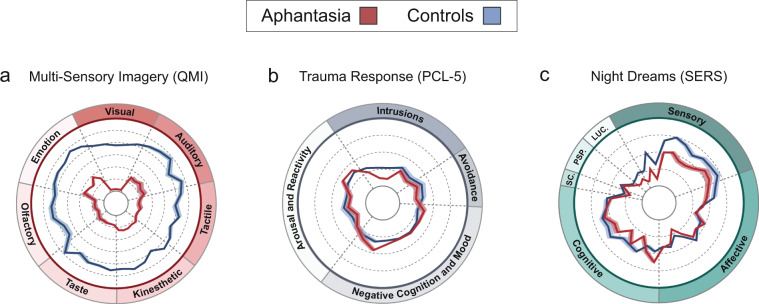
Group differences in visual imagery ability on scale sub-components. Radar plots for (**a**) multi-sensory imagery; (**b**) trauma response; and (**c**) dreaming scales (SC. = Spatial Complexity; PSP. = Perspective; LUC. = Lucidity). Concentric dashed circles represent raw scale scores for each scale (e.g. **a**; 1–7 Likert-type), with lowest possible item scores falling on innermost solid circle and highest possible item scores falling on outermost coloured circle; radial dashed lines denote item grouping for scale sub-components (e.g. **c**; Intrusions, Avoidance, Negative Cognition and Mood, Arousal and Reactivity); central coloured lines (red = aphantasia group, blue = control group 1) represent raw total group scores on individual scale items, with translucent shading denoting standard-deviation.

We first examined group differences in visual imagery vividness. As expected based on previous findings^9,10^, aphantasic participants rated their visual imagery ability on the VVIQ as being significantly lower (17.94 ± 0.223, with many (70%) scoring at floor, i.e. 16) compared to control group 1 (58.12 ± 0.888; Mann-Whitney *U* = 427.5, *p* < 0.0002, *r* = 0.87, BF_10_ = 1.41e^12^, 2-tailed; see Fig. 1 red section and Figure S1 in Supplementary Information; Fig. 1 depicts median-centered data with the aphantasia group denoted by red plots and control group 1 by blue plots throughout; Figures S1–5 show raw scale scores and distributions). This self-reported qualitative absence of visual imagery vividness was mirrored by significantly lower scores than controls on the object imagery component of the OSIQ (Mann-Whitney *U* = 372, *p* < 0.0002, *r* = 0.85, BF_10_ = 446,931.23, 2-tailed; see Fig. 1 red section and Fig. S1), which measures the perceived ability to use imagery as a cognitive tool in task-relevant scenarios. Our data also showed that individuals with aphantasia not only report being unable to visualise, but also report comparatively reduced imagery, on average, in all other sensory modalities (measured using the QMI), including auditory (*U* = 6,152, BF_10_ = 5.01e^11^), tactile (*U* = 4,473, BF_10_ = 4.90e^9^), kinesthetic (*U* = 5,151, BF_10_ = 1.04e^11^), taste (*U* = 3,069.5, BF_10_ = 4.82e^26^), olfactory (*U* = 3,439.5, BF_10_ = 2.73e^9^) and emotion (*U* = 6,670.5, BF_10_ = 4.81e^12^) domains (all Mann-Whitney U-tests, *p* < 0.0002, *r* = 0.65–.78, 2-tailed; see Fig. 2a and Fig. S1). It is noteworthy, however, that despite reporting a near total absence of visual imagery on the QMI (Mann-Whitney *U* = 620.5, *p* < 0.0002, *r* = 0.87, BF_10_ = 1.07e^9^, 2-tailed; see Fig. 2a) and significantly lower total QMI scores overall compared to controls (Mann-Whitney *U* = 1,868.5, *p* < 0.0002, *r* = 0.79, BF_10_ = 6.47e^12^, 2-tailed; see Fig. 1 red section, second panel from top), only 26.22% of aphantasic participants reported a complete lack of multi-sensory imagery altogether (rating each question in each QMI domain as “1: No sensory experience at all”). The remainder of our aphantasic sample (73.78%) reported some degree of imagery in non-visual sensory modalities (albeit significantly reduced compared to controls; see Fig. 1 red section, and Fig. 2a), suggesting potential sub-categories of aphantasia.

#### Memory results

Aphantasic individuals described a significantly lower ability to remember specific life events in general (Event Memory component of the SAM; Mann-Whitney *U* = 8,865, *p* < 0.0002, *r* = 0.58, BF_10_ = 4.68e^10^, 2-tailed; see Fig. 1 blue section) and reported almost no ability to generate visual sensory details when actively remembering past events (memory vividness on the EMIQ; Mann-Whitney *U* = 2,186.5, *p* < 0.0002, *r* = 0.81, BF_10_ = 1.01e^15^, 2-tailed; see Fig. 1 blue section and Fig. S2 in Supplementary Information) compared to participants in control group 1. However, these self-reported reductions in reliving events were not confined to the past, with aphantasics as a group also reporting a near total inability to imagine future hypothetical events in any sensory detail (Future Events component of the SAM; Mann-Whitney *U* = 7,469.5, *p* < 0.0002, *r* = 0.63, BF_10_ = 2.97e^10^, 2-tailed; see Fig. 1 blue section and Fig. S2). Self-reported factual (or semantic) memory, which is traditionally thought to provide a kind of ‘scaffold’ for event memories more widely^23^, also appeared to be lower in individuals unable to visualise compared to controls (Factual Memory component of the SAM; Mann-Whitney *U* = 18,601.5, *p* < 0.0002, *r* = 0.27, BF_10_ = 156,732.50, 2-tailed; see Fig. 1 blue section and Fig. S2), although this effect was of a lower magnitude than the memory reductions reported above (see Fig. 1 blue section and Table S7 in Supplementary Information). The fourth scale component of the SAM (Spatial Memory) is grouped with the Spatial Imagery component of the OSIQ in results below (see Spatial Ability Results).

#### Trauma response results

Our data did not directly support the hypothesis that visual imagery absence might protect aphantasic individuals from trauma symptomology in response to stressful life events, with the aphantasia group scoring comparatively to control group 1 on the PCL-5 overall (total PCL-5 scores; Mann-Whitney *U* = 27,515, *p* = 0.776, *r* = 0.01, BF_10_ = 0.12, 2-tailed; see Fig. 1 grey section and Figure S3 in Supplementary Information). An analysis of group differences on the four sub-components of this scale (Intrusions, Cognition and Mood, Avoidance, and Arousal) also revealed that there were no significant differences between the groups in reports of emotional arousal and reactivity associated with remembering stressful past events (Mann-Whitney *U* = 27,240, *p* = 0.924, *r* = 0.00, BF_10_ = 0.11, 2-tailed; see Fig. 2b and Fig. S3). Compared to participants with visual imagery, individuals with aphantasia appeared to report fewer recurrent and involuntary memory intrusions (Mann-Whitney *U* = 22,739, *p* = 0.002, *r* = 0.14, BF_10_ = 14.85, 2-tailed; see Fig. 2b and Fig. S3), lower engagement in avoidance behaviours (Mann-Whitney *U* = 23,164.5, *p* = 0.006, *r* = 0.13, BF_10_ = 2.13, 2-tailed; see Fig. 2b and Fig. S3), and greater negative changes in cognition and mood (Mann-Whitney *U* = 30,960, *p* = 0.008, *r* = 0.12, BF_10_ = 12.99, 2-tailed; see Fig. 2b and Fig. S3) in response to stressful life events, although none of these group differences survived Bonferroni correction for multiple comparisons, and effect size estimates were small (*r* = 0.12–.14; see Table S7 in Supplementary Information). Interestingly, however, Bayesian analyses indicated strong evidence in favour of group differences on the Intrusions (BF_10_ = 14.85) and Cognition and Mood (BF_10_ = 12.99) sub-scales of the PCL-5 reported above.

#### Day and night dream results

Here we found that although there was little evidence for or against (BF_10_ = 1.93 and BF_01_ = 0.518) a difference between groups in the reported frequency of day-dreaming (Mann-Whitney *U* = 23,001.5, *p* = 0.005, *r* = 0.13, 2-tailed, non-significant after Bonferroni correction; see Fig. 1 teal section and Figure S4 in Supplementary Information), aphantasic individuals did report experiencing significantly fewer night dreams than controls (Imaginal Process Inventory (IPI); Mann-Whitney *U* = 15,828.5, *p* < 0.0002, *r* = 0.37, BF_10_ = 4.24e^6^, 2-tailed; see Fig. 1 teal section and Fig. S4). Interestingly, the reported qualitative content of these night dreams also differed between groups as measured by the SERS. Dream reports for aphantasic individuals reinforce a model of aphantasia as being primarily characterised by sensory deficits (Sensory; Mann-Whitney *U* = 15,087.5, *p* < 0.0002, 0.38, BF_10_ = 5.46e^6^, 2-tailed) across all dream modalities (including olfactory, tactile, taste and auditory domains; see Fig. 2c and Fig. S4). Interestingly, aphantasic individuals also reported experiencing lower awareness and control during their dreams (Lucidity; Mann Whitney *U* = 19,473.0, *p* < 0.0002, *r* = 0.25, BF_10_ = 1902.01, 2-tailed). We found some evidence that the dreams aphantasic participants report are characterised by less vivid emotions (Affective; Mann Whitney *U* = 23,463.0, *p* = 0.013, non-significant after Bonferroni correction, *r* = 0.11, BF_10_ = 9.01, 2-tailed), and a less clear dreamer perspective (Perspective (PSP); Mann Whitney *U* = 22,070.5, *p* = 0.0004, *r* = 0.16, non-significant after Bonferroni correction, BF_10_ = 127.28, 2-tailed) compared to participants in control group 1. However, there were no significant differences between the aphantasia group and control group 1 in the experience of within-dream cognition (e.g. planning or remembering (Cognitive); Mann Whitney *U* = 24,592.0, *p* = 0.085, *r* = 0.08, BF_10_ = 1.05, 2-tailed) or the details of dreams’ spatial features (Spatial Complexity (SC); Mann Whitney *U* = 24,697.0, *p* = 0.092, *r* = 0.08, BF_10_ = 0.31, 2-tailed). Interestingly, the only question on the SERS for which aphantasics scored significantly higher than control group 1 participants was an item in the Cognitive domain (see Fig. 2c) which asks how much time participants spent thinking during their dreams (Mann-Whitney *U* = 34,401.5, *p* < 0.0002, BF_10_ = 3.53e^3^), which accords well with a reduction in the sensory qualities of dreams in aphantasia in favour of semanticised contents.

#### Spatial ability results

Aphantasic participants reported slightly lower spatial imagery ability on the spatial sub-component of the OSIQ when compared to control group 1 (Mann-Whitney U = 24,462, p = 0.001, *r* = 0.15, BF_10_ = 14.65, 2-tailed; see Fig. 1 purple section and Figure S5 in Supplementary Information), although this effect was not significant after Bonferroni correction. Additionally, the scores of aphantasic individuals on the Spatial Memory component of the SAM (which includes items measuring reported spatial navigation and naturalistic spatial memory ability) were not significantly different from controls (SAM; Mann-Whitney U = 24,720, p = 0.1, *r* = 0.08, BF_10_ = 0.23, 2-tailed; see Fig. 1 purple section and Fig. S5). These results demonstrate that overall there were no consistent differences in reported spatial abilities between aphantasic individuals and participants in control group 1.

### Control Group 2: Replication Analysis

Although control group 1 was age-matched, it featured a higher ratio of males to females (see Table S1) in contrast to our aphantasic sample (which comprised of more females than males). Some of the variables included in this study (such as spatial ability and PTSD susceptibility) are known to be influenced by gender. To address this potential issue, we ran a replication analysis with a second control group of first-year undergraduate psychology students using the same experimental design (their raw data is depicted alongside our original control group and aphantasic sample in Figures S1–5).

Participants in our second control group (*n* = 193) were recruited from a sample of undergraduate psychology students at the University of New South Wales, and completed the study in exchange for course credit. All participants in this second control group were included in final analysis (with no exclusions). These participants (mean age = 19.33 years, *SD* = 3.69, range = 17–55 years) were not matched on mean age with our aphantasic sample (mean age difference = 14.6 years, *p* < 0.01, BF_10_ = 1.23e^10^), but instead featured a higher proportion of females to males (73% females, compared to 48% females in our aphantasic sample and 35% females in control group 1 (our main control group of MTurk responders).

Comparison with this second control group revealed a similar overall pattern of group differences to those reported above, with few effect changes in imagery and memory related domains in particular (see Figures S1–5 and Tables S2–6 in Supplementary Information for a comparison of test results, as well as Table S7 for a comparison of effect sizes). Aphantasic participants scored significantly lower than control group 2 on all outcomes of the imagery and episodic memory questionnaires (all *p* < 0.0002, all *r* > 0.52, all BF_10_ > 1.42e^8^) with the exception of the factual memory component of the SAM (which was no longer significantly lower in aphantasics when compared to control group 2 after controlling for multiple comparisons; Mann-Whitney *U* = 21,496.0, *p* = 0.002, *r* = 0.14, BF_10_ = 3.196, 2-tailed).

Although our Bayes analysis suggested strong evidence for higher total PCL-5 scores in control group 2 compared to the aphantasic group (Mann-Whitney *U* = 21,464.0, *p* = 0.002, *r* = 0.14, BF_10_ = 12.76, 2-tailed), this effect was not significant after Bonferroni correction. However, the previously non-significant reduction in memory intrusions amongst aphantasic participants (compared to control group 1) was much stronger in this second group comparison (Mann-Whitney *U* = 15,134.5, *p* < 0.0002, *r* = 0.35, BF_10_ = 2.20e^7^, 2-tailed), as were lower reports of avoidance behaviours by aphantasic individuals compared to control group 2 (Mann-Whitney *U* = 18,494.5, *p* < 0.0002, *r* = 0.24, BF_10_ = 2494.67, 2-tailed). Compared to control group 2, however, aphantasic participants did not report significantly higher negative cognition and mood (Mann-Whitney *U* = 25,827.5, *p* = 0.97, *r* = 0.00, BF_10_ = 0.12, 2-tailed) or arousal (Mann-Whitney *U* = 25,517.0, *p* = 0.12, *r* = 0.07, BF_10_ = 0.34, 2-tailed) in response to stressful life events, in line with our main control group 1 comparisons.

Individuals with aphantasia reported significantly fewer night dreams than control group 2 (Mann-Whitney *U* = 17,156.0, *p* < 0.0002, *r* = 0.74, BF_10_ = 21,124.12, 2-tailed). However, they also reported significantly less frequent mind-wandering compared to participants in control group 2 (Mann-Whitney *U* = 19,271.5, *p* < 0.0002, *r* = 0.29, BF_10_ = 397.04, 2-tailed), in contrast to the results of our main analysis (which revealed no significant differences in mind-wandering reports between the aphantasic group and control group 1). Also in contrast to our initial dreaming results, aphantasic participants scored significantly lower than control group 2 on all components of the SERS (Sensory, Affective, Cognitive, Spatial Complexity, Perspective and Lucidity; all *p* < 0.0002, all *r* > 0.71, all BF_10_ > 1.56e^7^), including on some domains where there were no significant differences between aphantasic participants and age-matched participants in control group 1 (see Fig. S4 and Table S5). However, these findings may be partially explained by age-related decline in dream frequency and subjective recall^24^.

Lastly, there were no significant differences in reported spatial imagery ability on the OSIQ (Mann-Whitney *U* = 22,635.5, *p* = 0.03, *r* = 0.10, BF_10_ = 0.88, 2-tailed) or spatial navigation ability on the SAM (Mann-Whitney *U* = 23,760.5, *p* = 0.15, *r* = 0.07, BF_10_ = 0.23, 2-tailed) between the aphantasic group and control group 2, reinforcing our initial results as well as previous findings of preserved spatial (but not object) imagery in aphantasic participant samples^10,22^.

## Discussion

Here we found that individuals with aphantasia report significant reductions in sensory simulation across a range of volitional and non-volitional mental processes, and overall appear to demonstrate a markedly distinct pattern of cognition compared to individuals with visual imagery. Notably, aphantasic individuals reported significantly reduced imagery across all sensory modalities (and not just visual). However, only 26.22% of aphantasic participants reported a total absence of multi-sensory imagery altogether, raising important questions about the primary aetiology of aphantasia and suggesting possible sub-categories of aphantasia within a heterogeneous group. Aphantasic individuals’ episodic memory and ability to imagine future events were also reported to be significantly reduced compared to the two control populations. These findings attest to the recently established functional and anatomical overlap in brain networks supporting the flexible, constructive simulation of episodic events (whether they be real past events or hypothetical future events)^25^, and suggest that visual imagery may be an essential and unifying representational format potentiating these processes.

Interestingly, our data aligns with that of previous studies demonstrating unaffected spatial imagery abilities in aphantasia^10,22^, suggesting an important distinction between object imagery (low-level perceptual features of objects and scenes) and spatial imagery (spatial locations and relations in mental images)^26^. This distinction is indeed reflected at a neural level, with disparate brain pathways used for perceptual object processing and spatial locations, respectively^27^. Strikingly, cognitive differences in aphantasia were not limited to processes where visual imagery is typically deliberate and volitional, with aphantasic individuals in our study reporting significantly less frequent and less vivid instances of spontaneous imagery such as night dreams. These data suggest that any cognitive function (voluntary or involuntary^28^) involving a sensory visual component is likely to be reduced in aphantasic individuals, and it is this generalised reduction in the sensory simulation of complex events and scenes that is most striking in aphantasia.

This work used a large-sample design to investigate reports of altered cognitive processes as a function of visual imagery absence. However, due to the self-described nature of the phenomenon in our online sample, it is prudent to rule out alternative explanations for the between-group differences seen here. Some authors have appropriately highlighted that visual imagery absence does not always present congenitally, but may be acquired as an associated symptom of neurological damage or psychopathology^29^. As a result, it is arguable that some aspects of our results may be more parsimoniously attributed to underlying psychogenic factors. Whilst plausible, we do not believe the reports of our sample here are best explained by this account. Only 9 out of 267 (3%) participants in our aphantasic sample reported acquired imagery loss, with the majority of participants reporting having lacked visual imagery capacity since birth. Additionally, there were no significant differences between our aphantasic sample and our main control group in the number of participants reporting a history of mental illness, neurological condition, or head injury/trauma – in fact, significantly fewer aphantasic participants reported a history of stroke or history of epilepsy/seizures compared to participants in control group 1 (see Sample Characteristics in Method, and Table S1 in Supplementary Information).

Importantly, a supplementary within-group analysis also showed that there were no significant differences between aphantasic participants with or without a reported history of mental illness/psychopathology on any of our primary imagery, memory, dreaming, or spatial ability outcome variables, after controlling for multiple comparisons (see Table S8 in Supplementary Information). Furthermore, the only significant within-group differences that were revealed by this supplementary analysis (such as significantly higher scores on some PCL-5 components in aphantasic individuals with a mental illness history compared to those without; see Table S8) are differences we might expect to find as a function of psychopathology status in any sample population, given the target variables of interest and clinical scope of the scale. Considering these factors together, it is unlikely that our main results are best explained by acquired or associated symptoms of psychogenic causes such as mental illness or psychopathology.

Aphantasic participants in our study were compared with two independent control groups of participants with visual imagery on a range of self-reported cognitive outcomes. It is important to note that that neither of these control groups were perfectly matched on demographic characteristics with our aphantasic sample. In our main group comparison, the ratio of females to males was significantly higher in the aphantasic group (48%) than in control group 1 (35%), despite these groups being matched on mean age. In order to account for the potential influence of sample characteristics (including gender) in our main control group of MTurk participants, we conducted a replication analysis with a second control group of undergraduate students featuring a higher ratio of females to males (73%). This second control group, however, was significantly younger in mean age (19 years) compared to the aphantasic sample (34 years; see Table S1 in Supplementary Information).

Despite these demographic discrepancies, the results of our replication analysis with control group 2 revealed a remarkably similar pattern of between-group effects to our main analysis (see Tables S2–6 in Supplementary Information). Additionally, a majority of the significant changes to our results that did occur are congruent with established effects of age and gender on cognitive outcomes. For example, our finding that undergraduate participants reported significantly more frequent memory intrusions and avoidance behaviours than aphantasic participants in response to stressful life events may be explained by the typically higher prevalence of PTSD diagnosis and symptomatology amongst females^30^ (and younger females in particular^31^). Similarly, our replication analysis results suggested that aphantasic participants reported significantly fewer mind-wandering episodes and qualitatively impoverished dream phenomenology in additional SERS domains, but only in comparison to the comparatively younger undergraduate control group 2 and not when compared to the age-matched control group 1 (see Fig. S4 and Table S5 in Supplementary Information). This is a pattern of results which accords well with findings of age-related decline in spontaneous mind-wandering^32^ and subjective dream phenomenology^24^, respectively.

The few divergences in results between our main analysis (with control group 1) and replication analysis (with control group 2) are therefore largely consistent with previous research on the roles of age and gender in cognition. The overall equivalence of our results across these independent control group comparisons (despite demographic discrepancies between groups) suggests that our major findings are unlikely to be artifacts of sampling bias. Nevertheless, the interaction between demographic characteristics, imagery and cognition is potentially complex, and future research should overcome this limitation of our study design by implementing more precise selection criteria for matched control samples.

It is also important to highlight that our study assessed intergroup differences in cognition by using self-report outcomes which might be influenced by response biases. If aphantasic participants were motivated to respond in line with a self-identified lack of imagery (or even with perceived generalised cognitive deficits), for example, we would expect them to indiscriminately report at floor on all self-report measures of cognition, or at least on all scales measuring cognitive abilities typically thought to be reliant on visual imagery use. Their pattern of responses on some scales (particularly those measuring reported spatial abilities) suggests otherwise. On the SAM, aphantasic individuals reported no consistent reduction in spatial memory (or navigation) ability compared to controls, despite reporting memory deficits on all other components of this scale (see Fig. 1 blue and purple sections). More convincingly, aphantasic participants selectively reported deficits in object imagery but not spatial imagery on the OSIQ in our study, despite items corresponding to these two components being presented in randomised order within the same scale (see Fig. 1 blue and red sections). Lastly, previous research has shown that participants with self-described aphantasia do not just score at floor on self-report imagery questionnaires, but also exhibit lower scores than control group participants on a behavioural measure of sensory imagery strength which bypasses the need for self reports^10^, suggesting that response bias is not a most parsimonious explanation for presentations of self-described aphantasia. Demand characteristics cannot be unequivocally ruled out in the current study (as with any study of self-reports), and our findings should be validated with objective measures in future experiments. However, this study provides useful population-level data in order to highlight the veridical subjective differences that exist in a range of cognitive domains as a function of visual imagery absence.

There is strong theoretical impetus for future assessments of aphantasia, and our work highlights several areas of relevance that should be prioritised by future studies. For example, it is noteworthy that whilst the PCL-5 assesses one’s general response to stressful life events, it does not assess responses to recalling specific traumatic events^18^, nor does it have good measurement sensitivity for the imagery-based re-experiencing of such events. Whilst the overall pattern of our results suggests that aphantasic individuals do not appear to be markedly protected against all forms of trauma symptomatology, it may remain the case that they discernibly benefit from a reduced susceptibility to re-living these events in vivid sensory detail. Similarly, the self-report nature of our study does not allow for an objective, content-driven account of episodic memory function and phenomenology in aphantasia. Whilst some of the questions presented to participants on the EMIQ and on the SAM do ask them to report upon the visual experience of their memories, the distinction between remembering past life events and visually representing them is one which is not well delineated. There is therefore considerable scope for future experimental research to tease apart these separable component processes of episodic memory, and their relation to visual imagery absence in aphantasia.

Many other questions about aphantasia remain unanswered, including its longitudinal stability, the relative contribution of genetic and developmental factors to its aetiology, and its exact contribution to individual cognitive profiles. Our research presents an extended cognitive fingerprint of aphantasia and helps to clarify the role that visual imagery plays in wider consciousness and cognition. Visual imagery is a cognitive tool often taken for granted – an assumed precursor to our ability to think, learn, and simulate the world around us. This work demonstrates that such tools are not shared by everyone, and shines light on the rich but often invisible variations that exist in the internal world of the mind.

## Supplementary information

Supplementary Information.
