# Supplementary material for: A cognitive profile of multi-sensory imagery, memory and dreaming in aphantasia
Source: Sci Rep. 2020 Jun 22;10:10022. doi: 10.1038/s41598-020-65705-7 (PMC7308278; doi:10.1038/s41598-020-65705-7)
Supplement: Supplementary file 1 — Supplementary Information. [file 41598_2020_65705_MOESM1_ESM.pdf]

SUPPLEMENTARY INFORMATION

A cognitive profile of multi-sensory imagery, memory and dreaming in aphantasia

Alexei J. Dawes[1*,]

Rebecca Keogh[1],

Thomas Andrillon[1&2],

& Joel Pearson[1]

1. School of Psychology, The University of New South Wales, Sydney, New South Wales, Australia.
2. School of Psychological Sciences and Turner Institute for Brain and Mental Health, Monash University, Melbourne, Victoria, Australia.

***Corresponding Author**

E-mail: alexei.dawes@unsw.edu.au

Address: Future Minds Lab, School of Psychology, Level 16 Matthews Building, University of New South Wales, Sydney, Australia, 2052

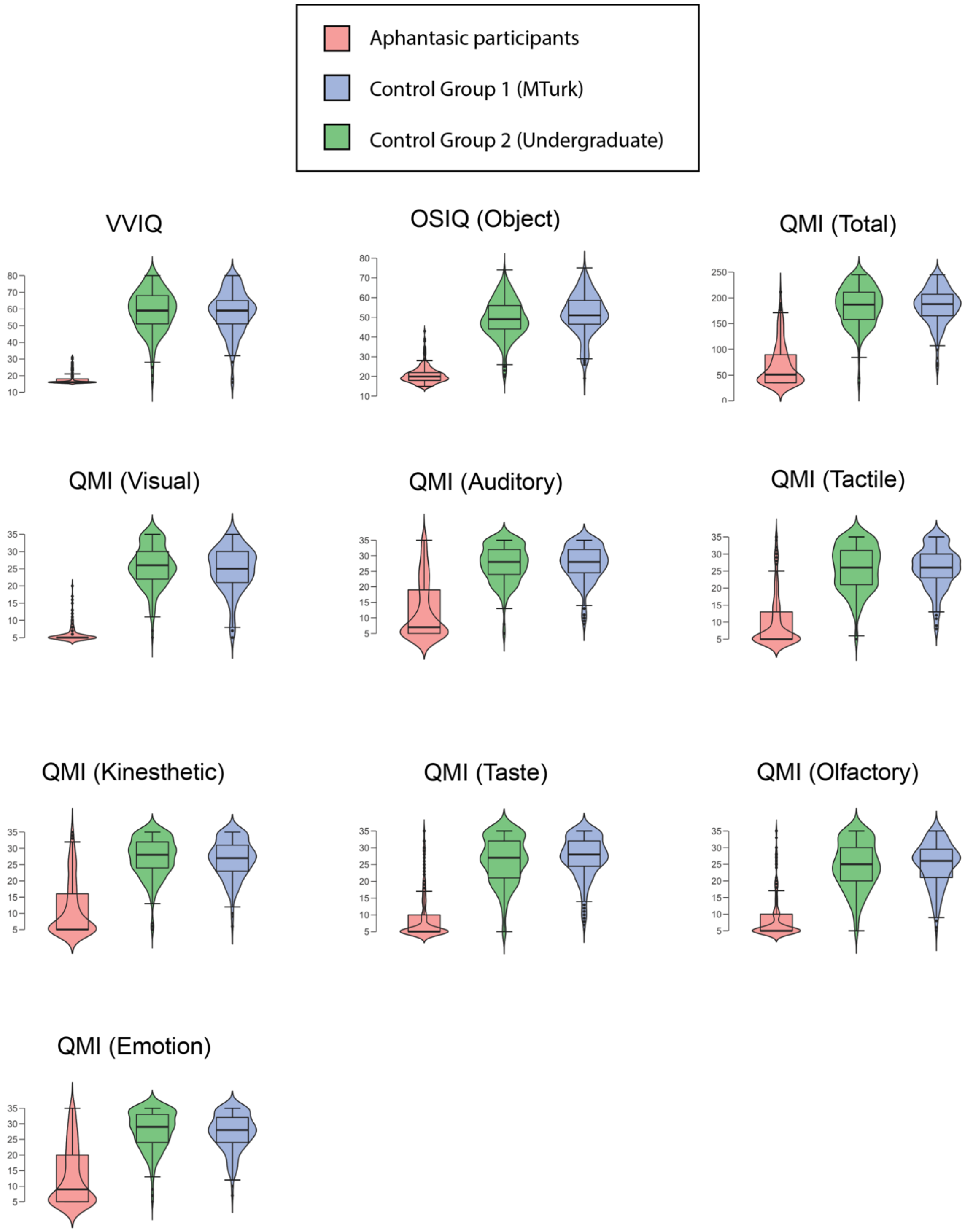

Figure S1. Average total scores as violin plots for imagery questionnaires split by imagery group: Aphantasic group (red), main control group (blue; MTurk responders), and second control group (green; undergraduate students). Error bars represent interquartile range, outliers are represented as black data points.

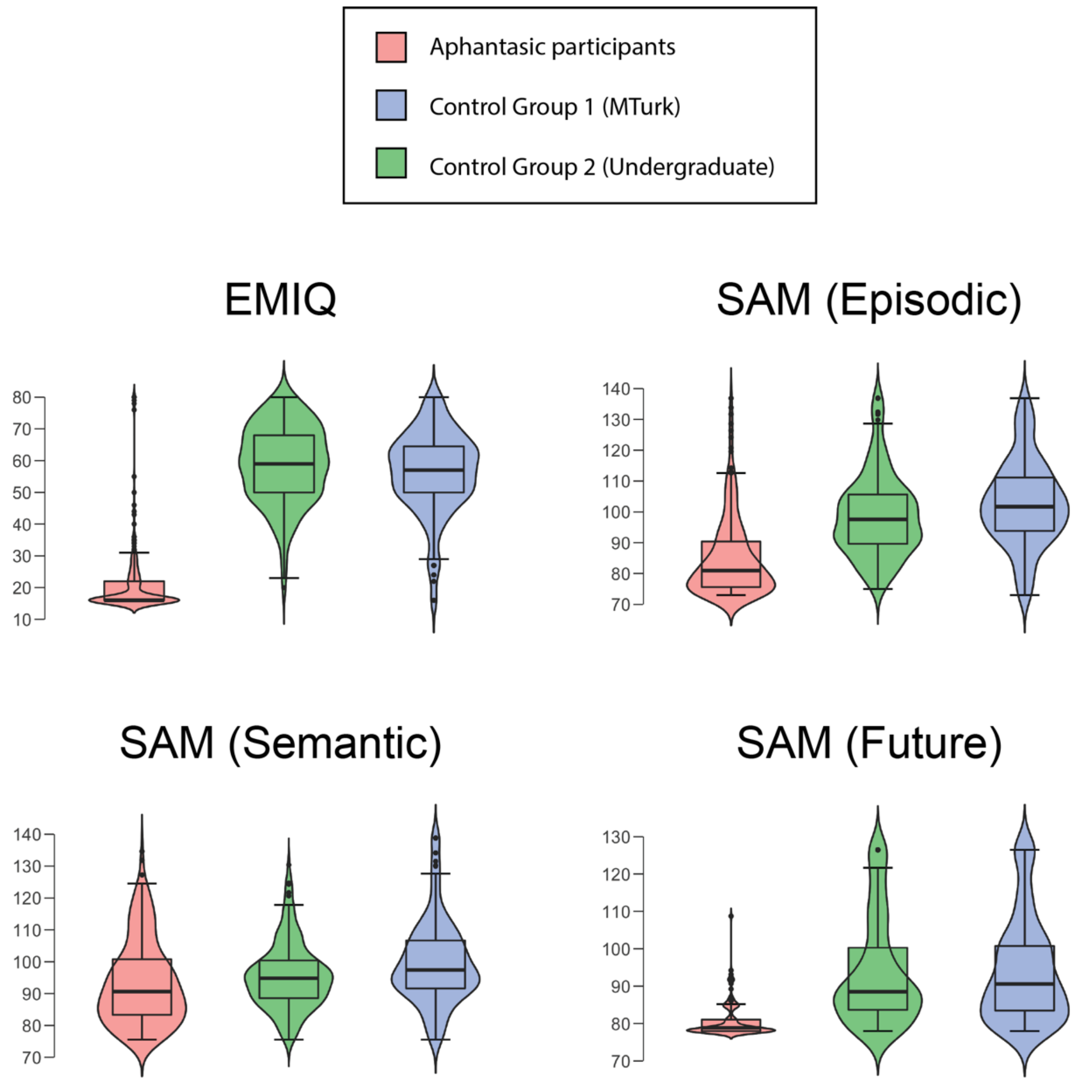

Figure S2. Average total scores as violin plots for memory questionnaires split by imagery group: Aphantasic group (red), main control group (blue; MTurk responders), and second control group (green; undergraduate students). Error bars represent interquartile range, outliers are represented as black data points.

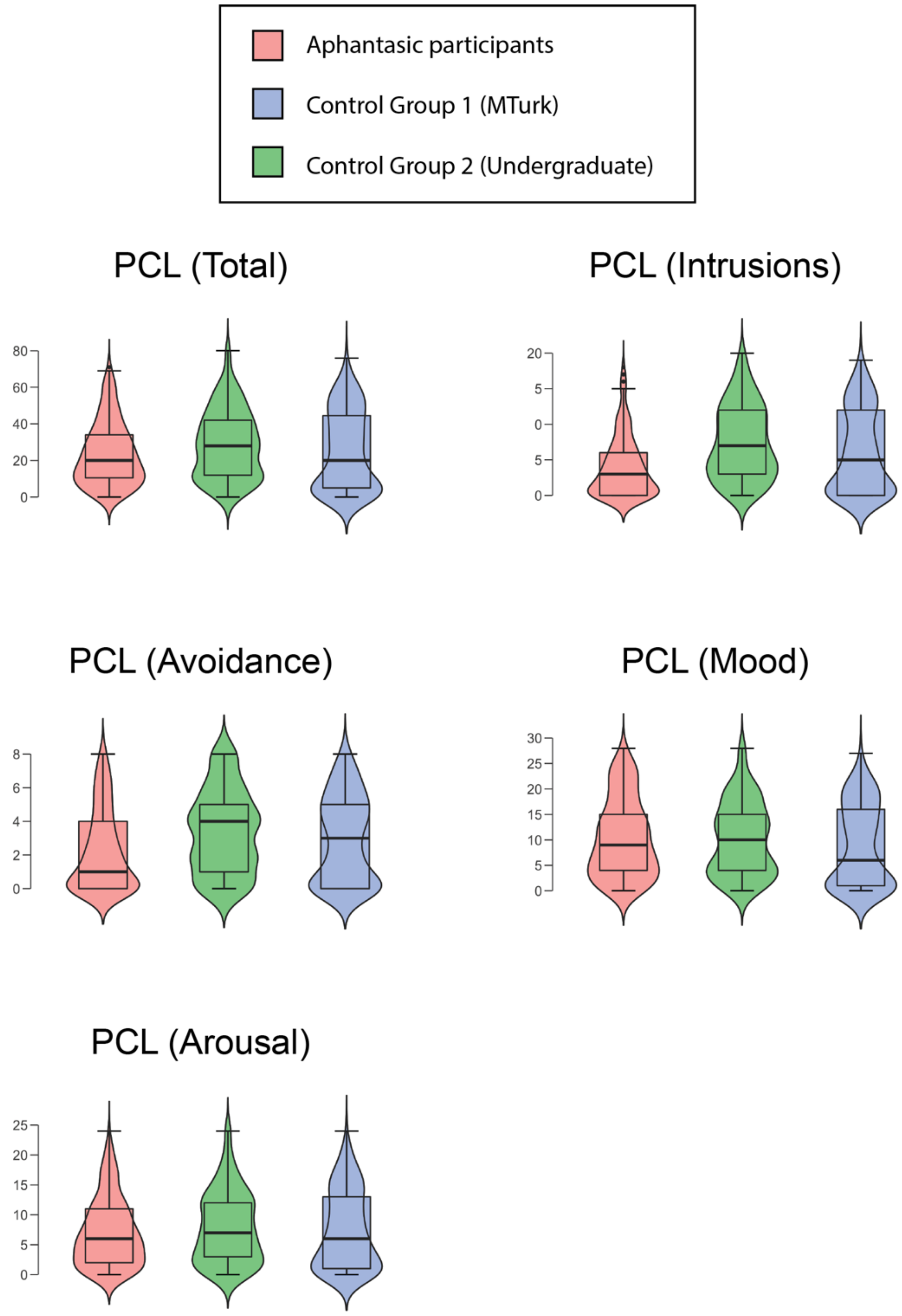

Figure S3. Average scores as violin plots for PCL-5 scale split by imagery group: Aphantasic group (red), main control group (blue; MTurk responders), and second control group (green; undergraduate students). Error bars represent interquartile range, outliers are represented as black data points.

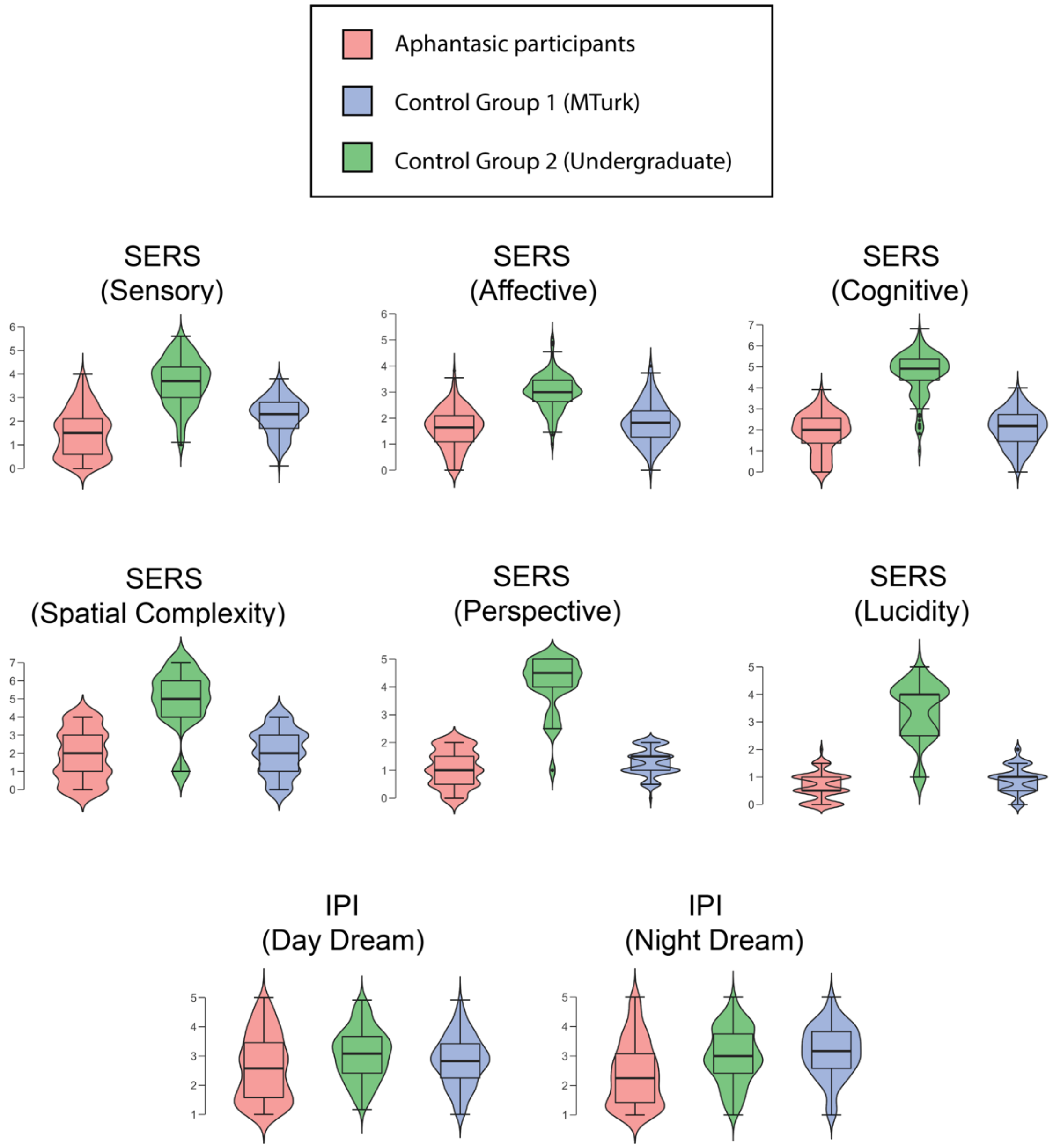

Figure S4. Average scores as violin plots for mind-wandering and dream questionnaires split by imagery group: Aphantasic group (red), main control group (blue; MTurk responders), and second control group (green; undergraduate students). Error bars represent interquartile range, outliers are represented as black data points.

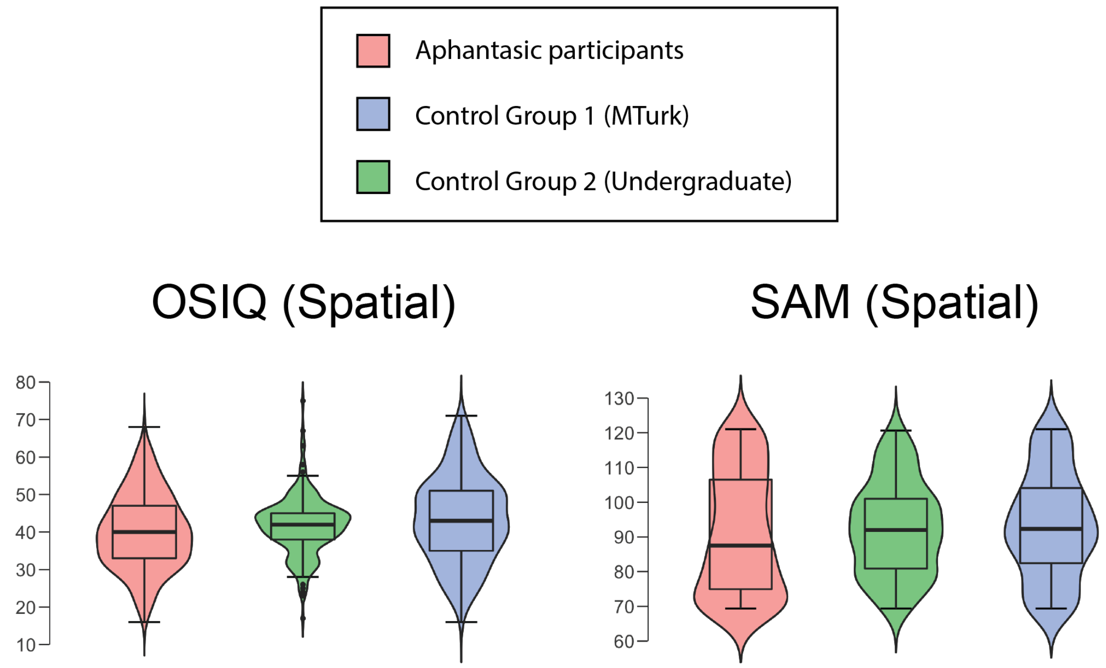

Figure S5. Average scores as violin plots for spatial questionnaires split by imagery group: Aphantasic group (red), main control group (blue; MTurk responders), and second control group (green; undergraduate students). Error bars represent interquartile range, outliers are represented as black data points.

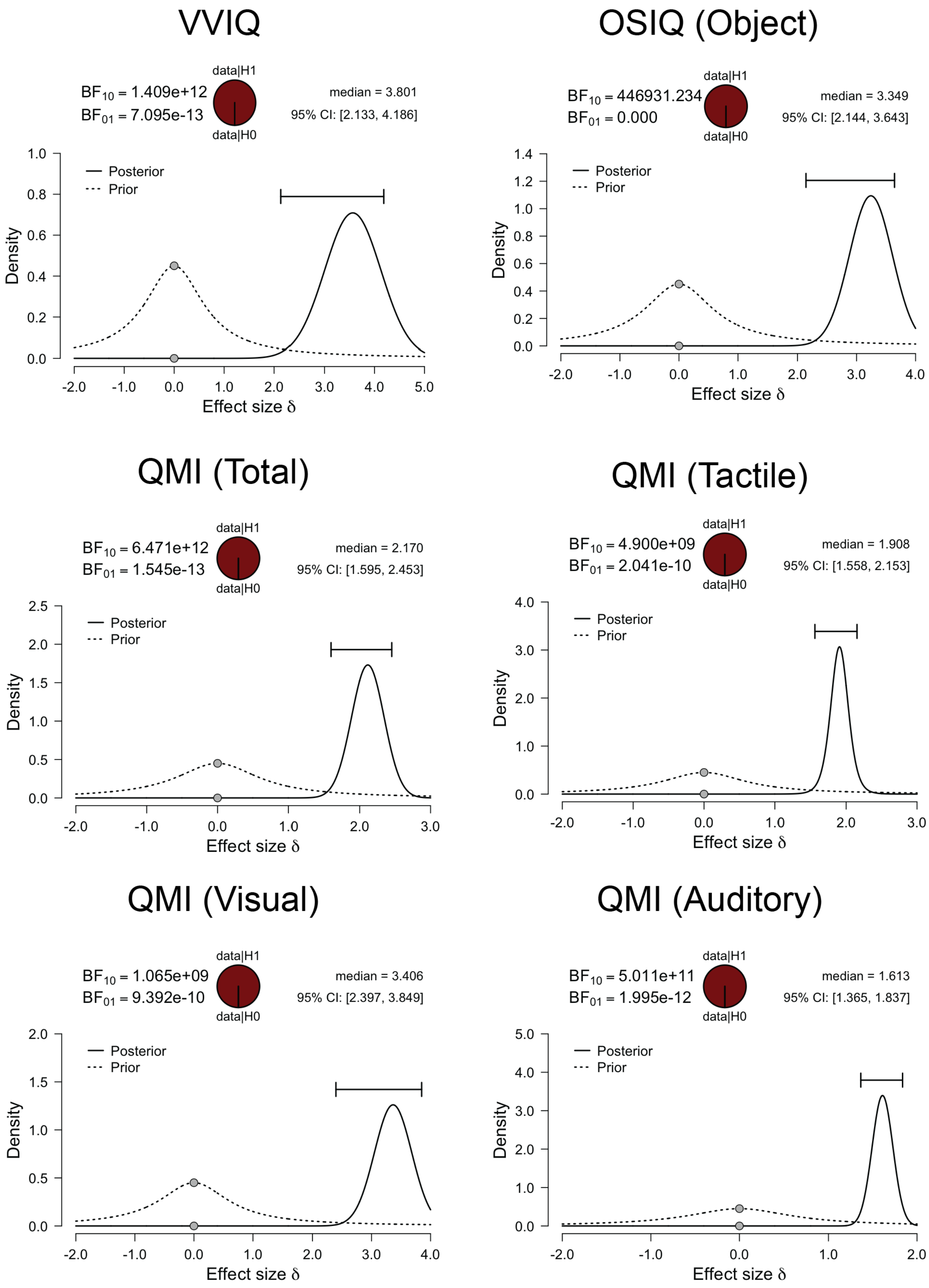

VVIQ
data|H1
BF10 = 1.409e+12
BF01 = 7.095e-13
data|H0
median = 3.801
95% CI: [2.133, 4.186]
Posterior
Prior
Density
Effect size δ
OSIQ (Object)
data|H1
BF10 = 446931.234
BF01 = 0.000
data|H0
median = 3.349
95% CI: [2.144, 3.643]
Posterior
Prior
Density
Effect size δ
QMI (Total)
data|H1
BF10 = 6.471e+12
BF01 = 1.545e-13
data|H0
median = 2.170
95% CI: [1.595, 2.453]
Posterior
Prior
Density
Effect size δ
QMI (Tactile)
data|H1
BF10 = 4.900e+09
BF01 = 2.041e-10
data|H0
median = 1.908
95% CI: [1.558, 2.153]
Posterior
Prior
Density
Effect size δ
QMI (Visual)
data|H1
BF10 = 1.065e+09
BF01 = 9.392e-10
data|H0
median = 3.406
95% CI: [2.397, 3.849]
Posterior
Prior
Density
Effect size δ
QMI (Auditory)
data|H1
BF10 = 5.011e+11
BF01 = 1.995e-12
data|H0
median = 1.613
95% CI: [1.365, 1.837]
Posterior
Prior
Density
Effect size δ

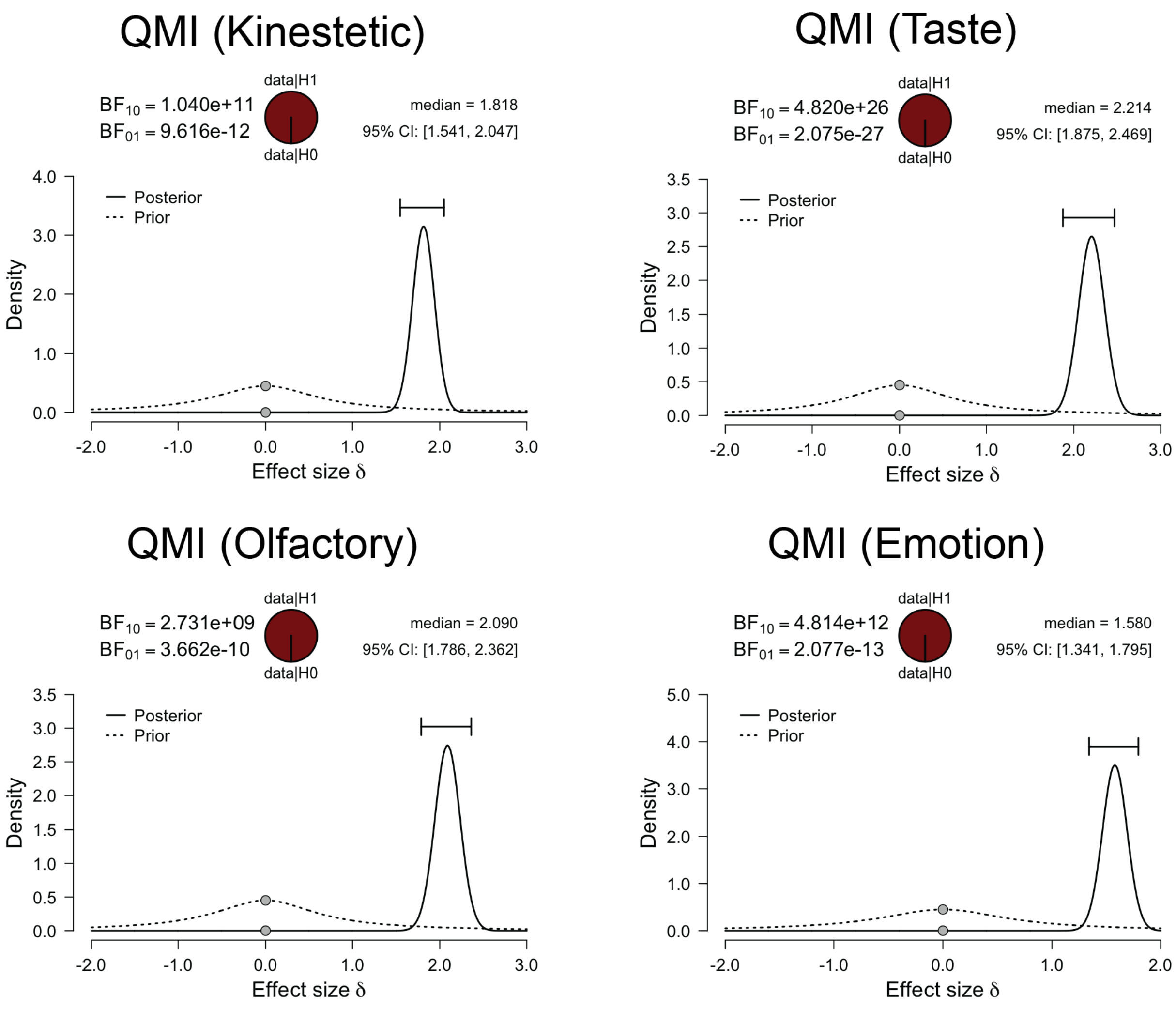

Figure S6. Prior and posterior plots for all imagery questionnaires. Data shows Mann-Whitney 2-sided t-tests, Cauchy = .707

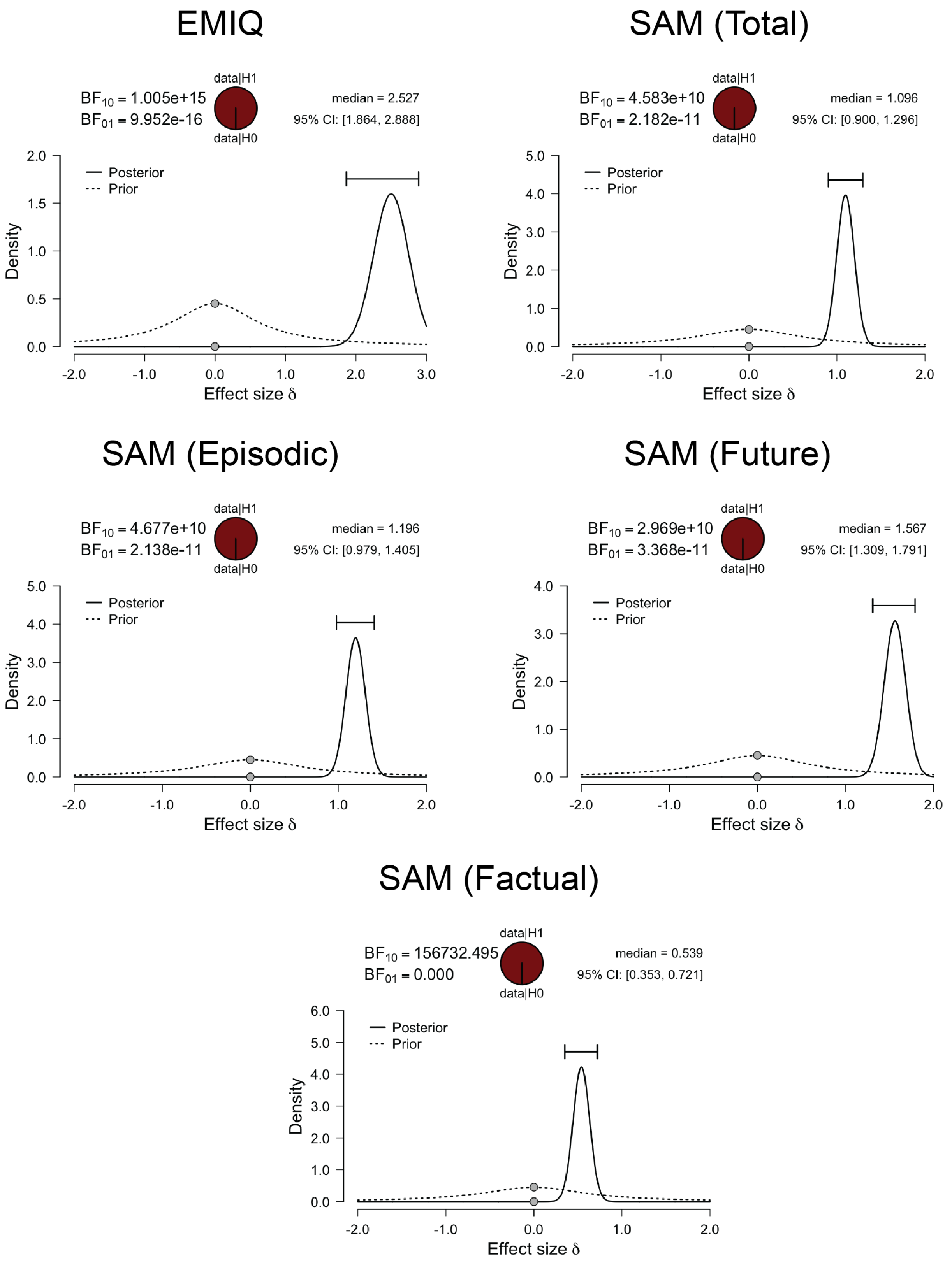

Figure S7. Prior and posterior plots for all memory questionnaires. Data shows Mann-Whitney 2-sided t-tests, Cauchy = .707

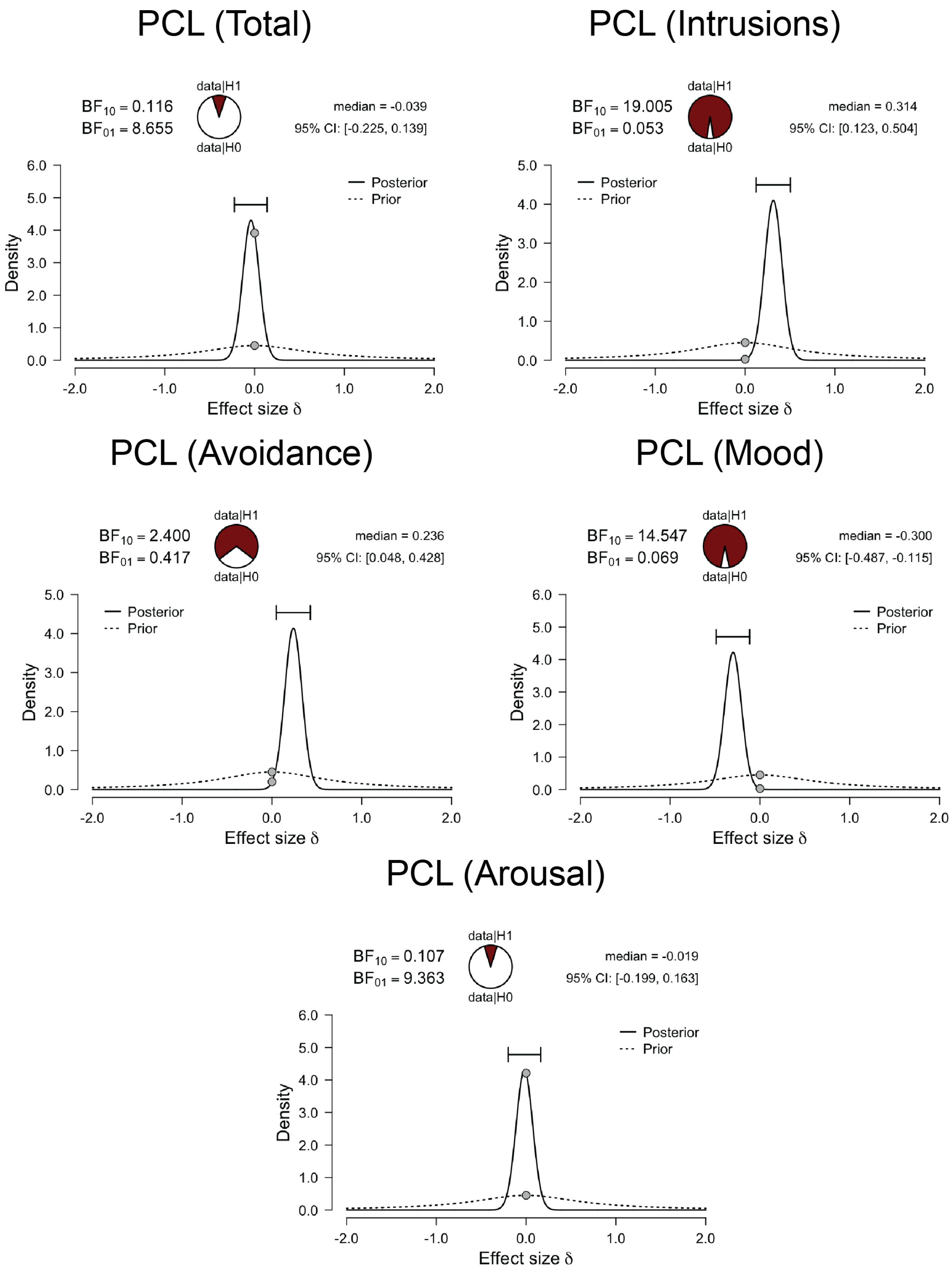

Figure S8. Prior and posterior plots for the PCL-5 scale. Data shows Mann-Whitney 2-sided t-tests, Cauchy = .707

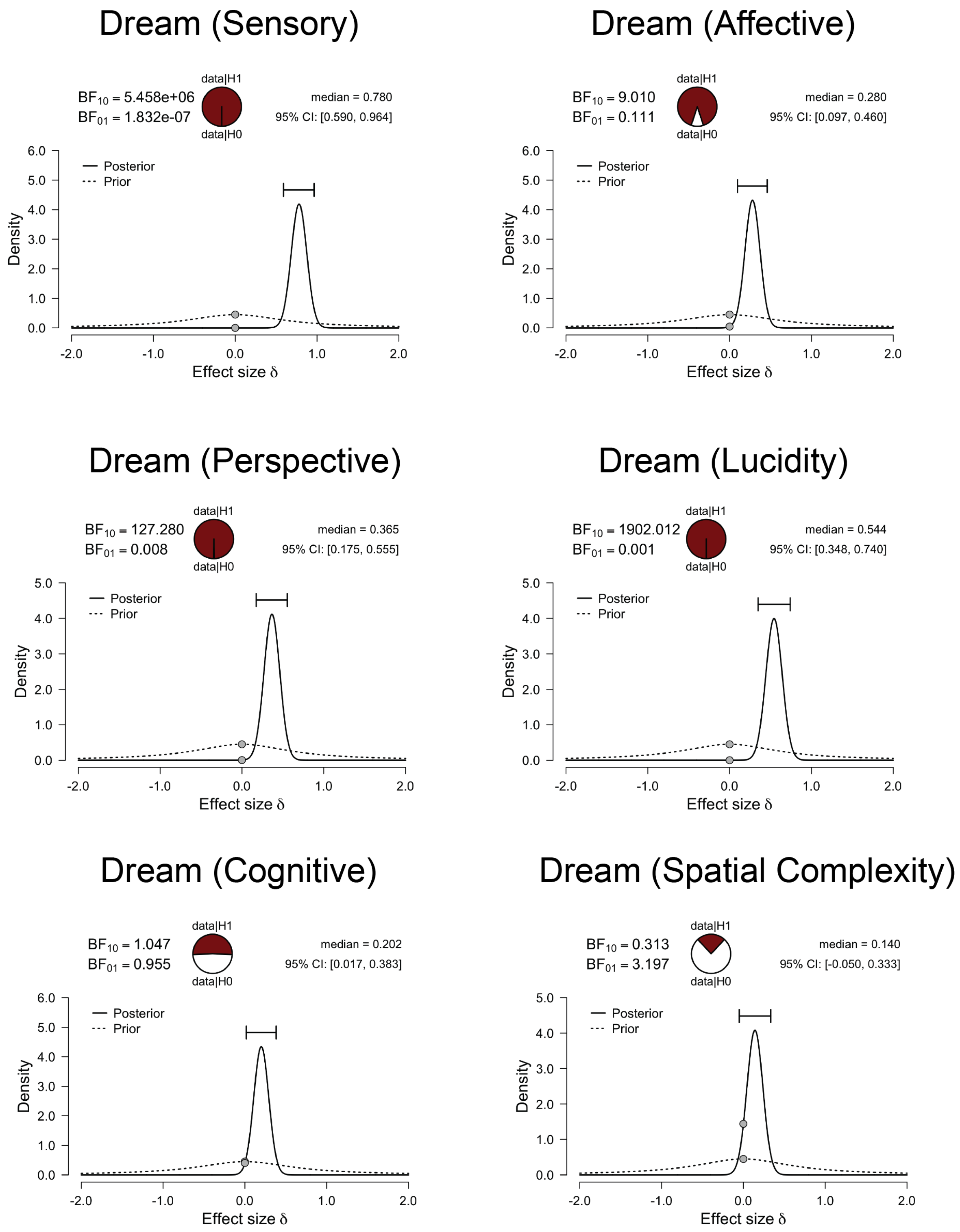

Dream (Sensory)
BF10 = 5.458e+06
BF01 = 1.832e-07
data|H1
data|H0
median = 0.780
95% CI: [0.590, 0.964]
Posterior
Prior
Density
Effect size δ
Dream (Affective)
BF10 = 9.010
BF01 = 0.111
data|H1
data|H0
median = 0.280
95% CI: [0.097, 0.460]
Posterior
Prior
Density
Effect size δ
Dream (Perspective)
BF10 = 127.280
BF01 = 0.008
data|H1
data|H0
median = 0.365
95% CI: [0.175, 0.555]
Posterior
Prior
Density
Effect size δ
Dream (Lucidity)
BF10 = 1902.012
BF01 = 0.001
data|H1
data|H0
median = 0.544
95% CI: [0.348, 0.740]
Posterior
Prior
Density
Effect size δ
Dream (Cognitive)
BF10 = 1.047
BF01 = 0.955
data|H1
data|H0
median = 0.202
95% CI: [0.017, 0.383]
Posterior
Prior
Density
Effect size δ
Dream (Spatial Complexity)
BF10 = 0.313
BF01 = 3.197
data|H1
data|H0
median = 0.140
95% CI: [-0.050, 0.333]
Posterior
Prior
Density
Effect size δ

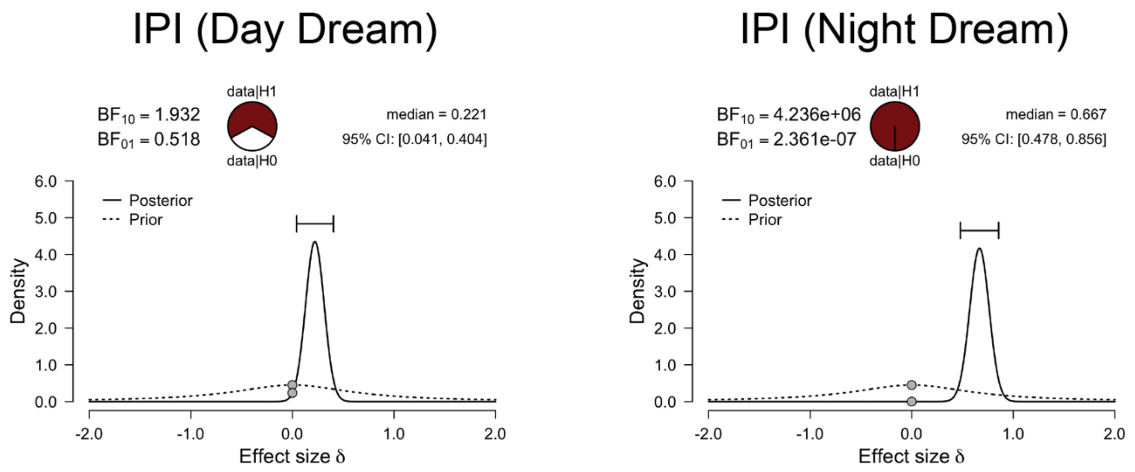

Figure S9. Prior and posterior plots for all mind-wandering and dream questionnaires. Data shows Mann-Whitney 2-sided t-tests, Cauchy = .707

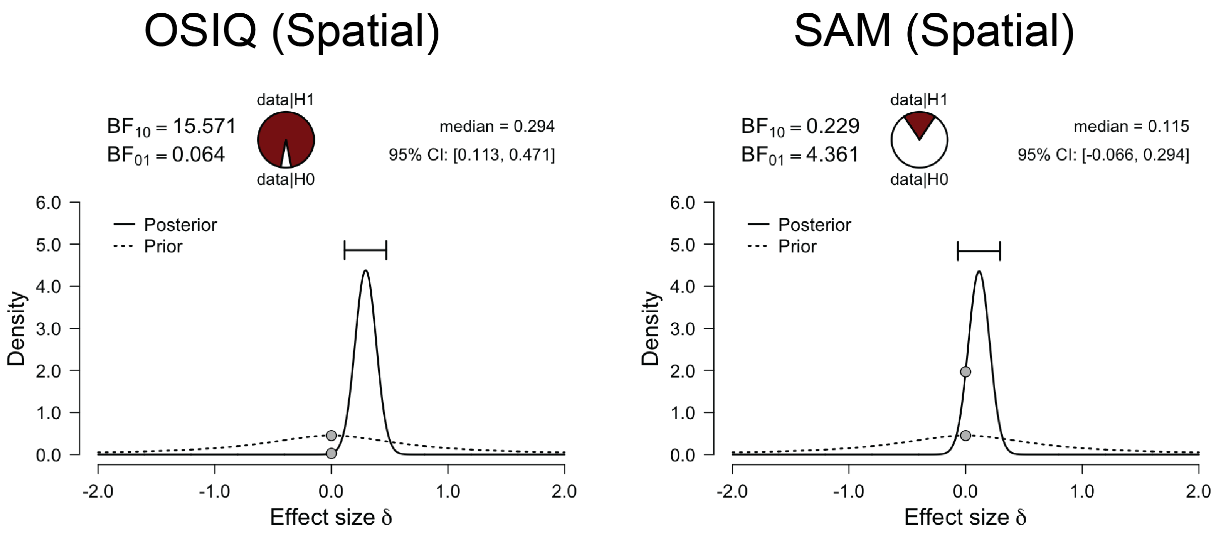

Figure S10. Prior and posterior plots for the Spatial Imagery component of the OSIQ, and the Spatial Memory component of the SAM. Data shows Mann-Whitney 2-sided t-tests, Cauchy = .707

Table S1

Table of demographic characteristics of study samples

	Aph[a] $N = 267$	C1[b] $N = 203$	C2[c] $N = 193$
**Age (SD) in years**	33.97 (12.44)	33.82 (9.33)	19.33 (3.69)
**Age Range in years**	17 – 75	20 – 70	17 – 55
**Gender (N, %)**			
Female	129 (48.31)	71 (34.98)	140 (72.54)
Male	109 (40.82)	131 (64.53)	53 (27.46)
Undisclosed	29 (10.86)	1 (.49)	–
**Ethnicity (N, %)**			
White/Caucasian	234 (87.65)	121 (59.61)	37 (19.17)
Asian	6 (2.25)	16 (7.88)	115 (59.56)
Indian	1 (.37)	41 (20.20)	14 (7.25)
African American	–	13 (6.40)	–
Hispanic	5 (1.87)	8 (3.94)	–
Arab/Middle East.	2 (.75)	–	8 (4.12)
Indigenous Aus.	–	–	1 (.52)
Other/Mixed	14 (5.24)	3 (1.48)	6 (3.12)
**English 1st Lang. (N, %)**			
Yes	220 (82.40)	181 (89.16)	145 (75.13)
No	47 (17.60)	22 (10.84)	48 (24.87)
**Clinical History (N, %)**			
Mental illness or Psychopathology	64 (23.97)	34 (16.75)	24 (12.44)
Epilepsy or seizures	3 (1.12)	17 (8.37)	1 (.52)
Neurological condition	11 (4.12)	14 (6.90)	24 (1.04)
Head injury or trauma	24 (8.99)	19 (9.36)	2 (1.04)
Stroke	2 (.75)	12 (5.91)	1 (.52)

[a] Aphantasic participants

[b] Control group 1 (MTurk participants)

[c] Control group 2 (Undergraduates)

Table S2

Table of imagery results by control group comparison

Variable (Scale)	Control group 1 (MTurk) vs. Aphantasics[a]					Control group 2 (undergrads) vs. Aphantasics[b]				
	Mean score (*C1*)	Mean score (*Aph*)	*U*	*p*	BF_{10}	Mean score (*C2*)	Mean score (*Aph)*	*U*	*p*	BF_{10}
Visual imagery (VVIQ)	58.12	17.94	427.5	.000*	1.41 e[12]	58.79	17.94	255.5	.000*	3.95e[10]
Object imagery (OSIQ)	51.65	20.70	372.0	.000*	446,931.23	49.40	20.70	452.0	.000*	4.08e[7]
Multi-sensory imagery total (QMI)	184.39	69.45	1,868.5	.000*	6.47e[12]	184.90	69.45	2,002.5	.000*	6.24e[18]
Visual imagery (QMI)	24.84	5.85	620.5	.000*	1.07e[9]	25.70	5.85	282.0	.000*	1.42e[8]
Auditory imagery (QMI)	27.47	12.13	6,152.5	.000*	5.01e[11]	27.32	12.13	6,185.5	.000*	4.04e[9]
Tactile imagery (QMI)	25.85	9.85	4,473.5	.000*	4.90e[9]	25.91	9.85	4,296.0	.000*	1.34e[10]
Kinesthetic imagery (QMI)	26.80	11.25	5,151.0	.000*	1.04e[11]	27.22	11.25	4,734.5	.000*	5.27e[14]
Taste imagery (QMI)	27.15	8.94	3,069.5	.000*	4.82e[26]	26.27	8.94	3,663.5	.000*	8.18e[11]
Olfactory imagery (QMI)	24.97	8.51	3,439.5	.000*	2.73e[9]	24.51	8.51	3,756.5	.000*	1.27e[9]
Emotion imagery (QMI)	27.31	12.92	6,670.5	.000*	4.81e[12]	27.96	12.92	5,999.5	.000*	3.09e[9]

Note: Data shows mean scores on each comparison variable (for directional interpretation), results of 2-sided Mann-Whitney tests (Mann-Whitney *U*, *p* value), and Bayes factors.

[a] Aphantasic participants compared to control group 1 (MTurk participants)

[b] Aphantasic participants compared to control group 2 (Undergraduates)

$^{*}p < 0.0002$ $\quad$ $^{\wedge}p < 0.01$

Table S3

Table of memory results by control group comparison

	Control group 1 (MTurk) vs. Aphantasics[a]					Control group 2 (undergrads) vs. Aphantasics[b]				
Variable (Scale)	Mean score (*C1*)	Mean score (*Aph*)	*U*	*p*	BF_{10}	Mean score (*C2*)	Mean score (*Aph)*	*U*	*p*	BF_{10}
Episodic Imagery (EMIQ)	56.43	20.83	2,186.5	.000*	1.01e[15]	58.99	20.83	1,488.5	.000*	3.997e[14]
Episodic Memory (SAM)	102.82	84.92	8,865.0	.000*	4.68e[10]	98.85	84.92	10,081.0	.000*	2.09e[8]
Future Imagery (SAM)	94.02	80.42	7,469.5	.000*	2.97e[10]	93.48	80.42	7,902.5	.000*	2.36e[13]
Factual Memory (SAM)	99.76	93.16	18,601.5	.000*	156,732.50	95.39	93.16	21,496.0	.002^	3.196

Note: Data shows mean scores on each comparison variable (for directional interpretation), results of 2-sided Mann-Whitney tests (Mann-Whitney *U*, *p* value), and Bayes factors.

[a] Aphantasic participants compared to control group 1 (MTurk participants)

[b] Aphantasic participants compared to control group 2 (Undergraduates)

$^{*}p < 0.0002$ $^{\wedge}p < 0.01$

Table S4

Table of trauma response results by control group comparison

	Control group 1 (MTurk) vs. Aphantasics[a]					Control group 2 (undergrads) vs. Aphantasics[b]				
Variable (Scale)	Mean score (*C1*)	Mean score (*Aph*)	*U*	*p*	BF_{10}	Mean score (*C2*)	Mean score (*Aph)*	*U*	*p*	BF_{10}
Trauma response total (PCL-5)	25.01	23.50	27,515.0	.776	.12	28.80	23.50	21,464.0	.002^	12.76
Memory intrusions (PCL-5)	6.01	3.98	22,739.0	.002^	14.85	7.42	3.98	15,134.5	.000*	$2.20e^7$
Avoidance (PCL-5)	2.91	2.21	23,164.5	.006^	2.13	3.36	2.21	18,494.5	.000*	2494.667
Negative cognition and mood (PCL-5)	8.81	10.34	30,960.0	.008^	12.99	10.17	10.34	25,827.5	.965	.108
Arousal and reactivity (PCL-5)	7.29	6.97	27,240.0	.924	.11	7.85	6.97	23,517.0	.109	.336

Note: Data shows mean scores on each comparison variable (for directional interpretation), results of 2-sided Mann-Whitney tests (Mann-Whitney *U*, *p* value), and Bayes factors.

[a] Aphantasic participants compared to control group 1 (MTurk participants)

[b] Aphantasic participants compared to control group 2 (Undergraduates)

$^*p < 0.0002$ $\quad$ $^\wedge p < 0.01$

Table S5
Table of dreaming and mind-wandering results by control group comparison

Variable (Scale)	Control group 1 (MTurk) vs. Aphantasics[a]					Control group 2 (undergrads) vs. Aphantasics[b]				
	Mean score (*C1*	Mean score (*Aph*)	*U*	*p*	BF_{10}	Mean score (*C2*)	Mean score (*Aph)*	*U*	*p*	BF_{10}
Day Dream (IPI)	2.84	2.60	23,001.5	.005^	1.93	3.04	2.60	19,271.5	.000*	397.038
Night Dream (IPI)	3.15	2.42	15,828.5	.000*	$4.24e^{6}$	2.99	2.42	17,156.0	.000*	21124.163
Dream Sensory (SERS)	2.23	1.49	15,087.5	.000*	$5.46e^{6}$	3.67	1.49	3,536.5	.000*	$6.49e^{8}$
Dream Affective (SERS)	1.83	1.60	23,463.0	.013	9.01	3.00	1.60	4,161.5	.000*	$3.85e^{8}$
Dream Cognitive (SERS)	2.06	1.89	24,592.0	.085	1.05	4.67	1.89	1,997.0	.000*	$1.56e^{7}$
Dream Spatial Complexity (SERS)	2.08	1.88	24,697.0	.092	0.31	4.95	1.88	4,173.5	.000*	$2.15e^{7}$
Dream Perspective (SERS)	1.29	1.08	22,070.5	.000^	127.28	4.11	1.08	858.0	.000*	$2.51e^{7}$
Dream Lucidity (SERS)	.92	.66	19,473.0	.000*	1902.01	3.38	.66	1,266.5	.000*	$2.44e^{7}$

Note: Data shows mean scores on each comparison variable (for directional interpretation), results of 2-sided Mann-Whitney tests (Mann-Whitney *U*, *p* value), and Bayes factors.

[a] Aphantasic participants compared to control group 1 (MTurk participants)

[b] Aphantasic participants compared to control group 2 (Undergraduates)

$^{*}p < 0.0002$ $\quad$ $^{\wedge}p < 0.01$

Table S6

Table of spatial imagery results by control group comparison

	Control group 1 (MTurk) vs. Aphantasics[a]					Control group 2 (undergrads) vs. Aphantasics[b]				
Variable (Scale)	Mean score (*C1*)	Mean score (*Aph*)	*U*	*p*	BF_{10}	Mean score (*C2*	Mean score (*Aph)*	*U*	*p*	BF_{10}
Spatial imagery (OSIQ)	43.46	40.14	22,462.0	.001^	14.65	41.74	40.14	22,635.5	.026	.883
Spatial memory (SAM)	93.12	90.95	24,720.0	.103	.23	92.36	90.95	23,760.5	.154	.229

Note: Data shows mean scores on each comparison variable (for directional interpretation), results of 2-sided Mann-Whitney tests (Mann-Whitney *U*, *p* value), and Bayes factors.

[a] Aphantasic participants compared to control group 1 (MTurk participants)

[b] Aphantasic participants compared to control group 2 (Undergraduates)

$^{*}p < 0.0002$ $\quad$ $^{\wedge}p < 0.01$

Table S7

Table of effect sizes by control group comparison

Variable (Scale)	Control group 1 (MTurk) vs. Aphantasics[a]		Control group 2 (Undergrad) vs. Aphantasics[b]	
	Z	*r*	*Z*	*r*
**Imagery**				
Visual imagery (VVIQ)	-18.914	0.8724	-18.784	0.8758
Object imagery (OSIQ)	-18.344	0.8461	-18.010	0.8397
Multi-sensory imagery total (QMI)	-17.328	0.7993	-16.921	0.7889
Visual imagery (QMI)	-18.963	0.8747	-18.962	0.8841
Auditory imagery (QMI)	-14.512	0.6694	-14.080	0.6565
Tactile imagery (QMI)	-15.789	0.7283	-15.554	0.7252
Kinesthetic imagery (QMI)	-15.234	0.7027	-15.148	0.7063
Taste imagery (QMI)	-16.887	0.7789	-16.158	0.7534
Olfactory imagery (QMI)	-16.678	0.7693	-16.128	0.7520
Emotion imagery (QMI)	-14.100	0.6504	-14.151	0.6598
**Memory**				
Episodic Imagery (EMIQ)	-17.517	0.8080	-17.700	0.8253

Episodic Memory (SAM)	-12.509	0.5770	-11.152	0.5200
Future Imagery (SAM)	-13.667	0.6304	-12.926	0.6027
Factual Memory (SAM)	-5.827	0.2688	-3.035	0.1415
**Trauma Response**				
Trauma response total (PCL-5)	.284	0.0131	-3.058	0.1426
Memory intrusions (PCL-5)	-3.026	0.1396	-7.593	0.3540
Avoidance (PCL-5)	-2.757	0.1272	-5.244	0.2445
Negative cognition and mood (PCL-5)	2.652	0.1223	.044	0.0021
Arousal and reactivity (PCL-5)	.096	0.0044	-1.601	0.0746
**Day Dream**				
Day Dream (IPI)	-2.811	0.1297	-4.616	0.2152
Night Dream (IPI)	-7.731	0.3566	-6.120	0.2853
**Night Dream**				
Dream Sensory (SERS)	-8.240	0.3801	-15.802	0.7368
Dream Affective (SERS)	-2.496	0.1151	-15.360	0.7162

Dream Cognitive (SERS)	-1.721	0.0794	-16.896	0.7878
Dream Spatial Complexity (SERS)	-1.685	0.0777	-15.502	0.7228
Dream Perspective (SERS)	-3.551	0.1638	-17.832	0.8314
Dream Lucidity (SERS)	-5.454	0.2516	-17.641	0.8225
**Spatial Ability**				
Spatial imagery (OSIQ)	-3.182	0.1468	-2.226	0.1038
Spatial memory (SAM)	-1.633	0.0753	-1.425	0.0664

Note: Data shows standardized *Z* scores from Mann-Whitney *U* non-parametric tests, and effect sizes *r* (for derivation, see Statistical Analyses in Method).

[a] Aphantasic participants compared to control group 1 (MTurk participants)

[b] Aphantasic participants compared to control group 2 (Undergraduates)

Table S8

Table of within-group differences as a function of mental illness history in aphantasic sample

Variable (Scale)	Mean score (Aph no MIH [b]) ***N* = 203**	Mean score (Aph with MIH [a]) ***N* = 64**	*U*	*p*
**Imagery**				
Visual imagery (VVIQ)	17.87	18.16	6,766.5	.535
Object imagery (OSIQ)	20.46	21.45	7,234.5	.168
Multi-sensory imagery total (QMI)	68.19	73.47	6,776.5	.599
Visual imagery (QMI)	5.87	5.77	6,180.5	.434
Auditory imagery (QMI)	12.14	12.13	6,400.5	.851
Tactile imagery (QMI)	9.45	11.11	6,810.5	.518
Kinesthetic imagery (QMI)	11.31	11.06	6,357.5	.783
Taste imagery (QMI)	8.76	9.52	6,876.0	.413
Olfactory imagery (QMI)	8.17	9.61	6,932.5	.337
Emotion imagery (QMI)	12.49	14.28	7,099.5	.247
**Memory**				
Episodic Imagery (EMIQ)	20.44	22.06	6,734.0	.609

Episodic Memory (SAM)	85.22	84.00	5,734.5	.157
Future Imagery (SAM)	80.46	80.31	6,074.5	.405
Factual Memory (SAM)	93.17	93.13	6,592.0	.859
**Trauma Response**				
Trauma response total (PCL-5)	20.20	33.97	9,329.0	.000*
Memory intrusions (PCL-5)	3.44	5.70	8,040.0	.004^
Avoidance (PCL-5)	1.88	3.27	8,279.0	.001^
Negative cognition and mood (PCL-5)	8.88	15.00	9,416.0	.000*
Arousal and reactivity (PCL-5)	6.01	10.00	9,118.0	.000*
**Day Dream**				
Day Dream (IPI)	2.47	3.00	8,206.0	.001^
Night Dream (IPI)	2.33	2.68	7,838.0	.013
**Night Dream**				
Dream Sensory (SERS)	1.45	1.64	7,089.0	.271
Dream Affective (SERS)	1.57	1.71	7,317.0	.127

Dream Cognitive (SERS)	1.88	.95	6,528.0	.953
Dream Spatial Complexity (SERS)	1.79	2.16	7,448.0	.071
Dream Perspective (SERS)	1.02	1.26	7,801.0	.013
Dream Lucidity (SERS)	.65	.70	6,830.0	.518
**Spatial Ability**				
Spatial imagery (OSIQ)	40.32	39.56	6,498.5	.996
Spatial memory (SAM)	91.63	88.78	5.975.5	.334

Note: Data shows results of 2-sided Mann-Whitney tests (Mann-Whitney *U*, *p* value) comparing aphantasic participants with mental illness history to aphantasic participants without mental illness history (split sample, within-group analysis).

[a] Aphantasic participants <u>with</u> a reported history of mental illness or psychopathology

[b] Aphantasic participants with <u>no</u> reported history of mental illness or psychopathology

$^{*}p < 0.0002$ $\quad$ ^$p < 0.01$
